# Metagenomic Characterization of Resistance Genes in Deception Island and Their Association with Mobile Genetic Elements

**DOI:** 10.3390/microorganisms10071432

**Published:** 2022-07-15

**Authors:** Andrés Santos, Felipe Burgos, Jaime Martinez-Urtaza, Leticia Barrientos

**Affiliations:** 1 Laboratorio de Biología Molecular Aplicada, Centro de Excelencia en Medicina Traslacional, Avenida Alemania 0458, Temuco 4810296, Chile; f.burgos02@ufromail.cl; 2 Institut de Biotecnologia i de Biomedicina, Universitat Autònoma de Barcelona, 08193 Cerdanyola del Vallès, Spain; 3 Departament de Genètica i de Microbiologia, Facultat de Biociéncies, Universitat Autònoma de Barcelona, 08193 Bellaterra, Spain; jaime.martinez.urtaza@uab.cat; 4 Núcleo Científico y Tecnológico en Biorecursos (BIOREN), Universidad de La Frontera, Avenida Francisco Salazar 01145, Temuco 4811230, Chile

**Keywords:** Antarctic soils, phages, plasmids, pristine environments

## Abstract

Antibiotic resistance genes (ARGs) are undergoing a remarkably rapid geographic expansion in various ecosystems, including pristine environments such as Antarctica. The study of ARGs and environmental resistance genes (ERGs) mechanisms could provide a better understanding of their origin, evolution, and dissemination in these pristine environments. Here, we describe the diversity of ARGs and ERGs and the importance of mobile genetic elements as a possible mechanism for the dissemination of resistance genes in Antarctica. We analyzed five soil metagenomes from Deception Island in Antarctica. Results showed that detected ARGs are associated with mechanisms such as antibiotic efflux, antibiotic inactivation, and target alteration. On the other hand, resistance to metals, surfactants, and aromatic hydrocarbons were the dominant ERGs. The taxonomy of ARGs showed that *Pseudomonas*, *Psychrobacter*, and *Staphylococcus* could be key taxa for studying antibiotic resistance and environmental resistance to stress in Deception Island. In addition, results showed that ARGs are mainly associated with phage-type mobile elements suggesting a potential role in their dissemination and prevalence. Finally, these results provide valuable information regarding the ARGs and ERGs in Deception Island including the potential contribution of mobile genetic elements to the spread of ARGs and ERGs in one of the least studied Antarctic ecosystems to date.

## 1. Introduction

Antimicrobial resistance is considered one of the most critical health threats of the 21st Century, where one of the main concerns is the dissemination of resistant pathogens and resistance mechanisms. The aforementioned has led to an urgent need to understand the origin, evolution, and spread of antibiotic resistance in the environment [1,2]. In this context, a current preoccupation is that resistance genes and mechanisms are undergoing especially rapid geographical spread in several ecosystems [3]. Thus, several studies have proposed the anthropogenic impact, horizontal gene transfer, and mobile genetic elements as drivers for the introduction and dissemination of these resistance mechanisms in microbial communities [4,5,6]. However, it is important to highlight that bacteria, and other microorganisms, have used antibiotics like competitive mechanisms for millions of years. Therefore, different resistance genes such as antibiotics and environmental resistance genes can be considered “background resistance” in all environments. Nowadays, there is no definitive answer when trying to distinguish background resistance from anthropogenic-caused resistance. Some authors suggest that interspecies competition and vertical inheritance could explain the presence of ARGs in pristine ecosystems. In this context, studies have shown that most of the ARGs are rarely associated with mobile genetic elements. Therefore, they can hardly be transferred horizontally between bacterial hosts without antibiotic stress [7] Thus, these vertical inherited ARGs could be considered a mechanism of ancient antibiotic resistance dissemination, while more recent resistance mechanisms in pristine environments could be associated with mobile genetic elements [8,9]. In this regard, culture-dependent studies have shown that Antarctic peninsula soils possess a diverse resistome and demonstrated the possible influence of mobile genetic elements in this diversity [10,11]. Thus, studying resistance genes in pristine environments could be considered a valuable source of information to address fundamental research questions regarding ARGs and ERGs dissemination.

There is evidence of antibiotic resistance genes (ARGs) even in the most pristine environments, such as the Antarctic territory, which is one of the most extreme and isolated environments on earth. Antarctica is characterized by a long history of isolation from the anthropogenic activity and harsh conditions such as extremely low temperatures, high radiation, rapid drainage, and limited organic nutrients. Nowadays, there is evidence that efflux pumps, bypass mechanisms, target modification, and target inactivation are the most commonly detected ARG types from soils on King George Island [8].

Although there is evidence of ARGs in the Antarctic continent, there are few studies carried out on Deception Island (62°57′ S, 60°38′ W) which is one of the less explored and more extreme zones in the Antarctic. This island is an active volcano that is part of the “South Shetlands”. It is about a diameter of 15 Km that was formed about 10,000 years ago [12]. Its geothermal activity and fumaroles have caused numerous eruptions that have modified the soil composition of the island, where high sulfate and oxidant levels are the main soil components [13,14], making Deception Island a poly-extreme environment. The aforementioned becomes relevant since co-resistance between heavy metals, harsh environmental conditions, and antibiotics have been demonstrated in minimally human-impacted environments [14,15,16]. Thus, Deception Island could be considered an invaluable source of information for studying ARGs and ERGs since the poly-extreme conditions of Deception Island may have favored the fixation of ARGs and ERGs in the microbial community. Furthermore, studying their dissemination mechanisms could allow us to obtain new insights into the origin and importance of resistance genes for microbial communities from pristine environments. Therefore, in the present study, we describe for the first time the diversity of ARGs and ERGs in Deception Island soil microbial communities and their possible dissemination mechanisms in the ecosystem.

## 2. Materials and Methods

### 2.1. Sample Collection and Site Description

**Figure 1:**
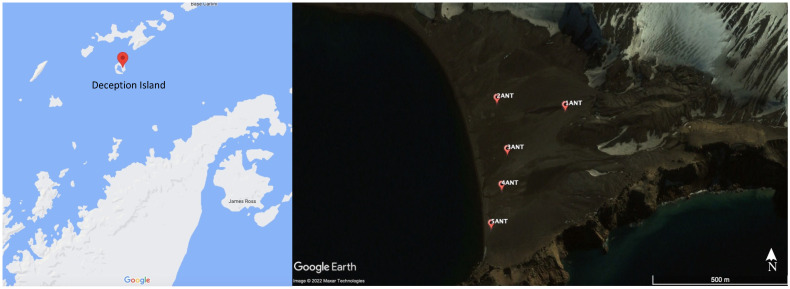
Map of Deception Island showing the location of sample sites. The map was taken using Google Maps (https://www.google.cl/maps; accessed on 1 April 2022) and sites are shown with red markers.

The samples used in this study were collected from the soils of Deception Island (Figure 1 and Table 1) during the 51st Scientific Antarctic Expedition from the Chilean Antarctic Institute. It is important to note that this expedition was in the summer season. Samples were aseptically taken from the upper 5 cm layer of soil and deposited in sterile Falcon tubes. The temperature and pH were measured in situ. Afterward, samples were kept in ice, transported to the laboratory, and frozen at −80 °C. In addition, nutrient determination (C and N) was carried out using an elemental analyzer Eurovector EA 3000, Milano, Italy.

### 2.2. DNA Extraction and Sequencing

Metagenomic DNA was extracted from five soil samples by a modified method [17] using the DNAeasy Powersoil^®^ Kit (QIAGEN, Hilden, Germany). Briefly, samples were heated at 70 °C for 10 min and immediately frozen at −80 °C for 10 min. Then, enzymatic lysis was carried out with Lysozyme (1:100) and proteinase K (20 mg/mL) (Thermo Fisher Scientific, Waltham, MA, USA). Afterward, the DNA extraction process was performed according to the DNAeasy Powersoil^®^ Kit manufacture indications. Finally, DNA was quantified using the One DNA QuantiFluor^®^ ONE dsDNA System (Promega, Madison, WI, USA) on a Quantus fluorometer. Afterward, DNA was sequenced under a metagenomic approach on a Novaseq6000 Sequencer using a LITE Library 150 bp PE at Earlham Institute (Norwich, UK). Metagenenomes nucleotide sequences have been deposited into GenBank under the bioproject accession PRJNA798087.

### 2.3. Bioinformatic Data Analysis

#### 2.3.1. Metagenomic Assembly

Metagenomic reads were quality trimmed using TrimGalore v0.6.0 [18] following default parameters and applying a q28 for quality score. High-quality reads were assembled using SPAdes v13.3 [19] with default parameters for the metagenome module (metaspades.py) using a k-mer length of 21, 33, 55, 99, and 127.

#### 2.3.2. Resistance Genes Identification

(a) Antibiotic resistance genes: Identification of clinical ARGs within the metagenomes was carried out with the Deeparg v2.0 Tool [20] using a concatenated database including the CARD [21] and ARBD databases [22] which are validated databases for ARGs. An identity cutoff of 60% was used to filter annotations and conduct further analyses. In this study, we have calculated the relative abundances of ARGs as the total number of ARGs per sample divided by the total number of Prodigal predicted genes as described by Van Goethem et al., 2018 [3].
(b) Environmental resistance genes: Identification of ERGs in the metagenomes was done using a custom pipeline. Briefly, a gene prediction was carried out using Prodigal v2.6.3.8 [23]. Subsequently, Hmmscan v3.3 was used to identify the ERG-Like genes in coding regions using the SargFam v2.0 database. Afterward, Diamond v0.9.14 [24] was used for ERGs annotation using the BacMet v2.0 database [25]. An identity cutoff of 60% was used to filter annotations and carry out further analyses. Finally, plots and ARGs and ERGs diversity analyses were made using the MicrobiomeAnalyst tool [26]. In this study, we have calculated the relative abundances of ERGs as the total number of ERGs per sample divided by the total number of Prodigal predicted genes as described by Van Goethem et al., 2018 [3].

#### 2.3.3. Taxonomy of Resistance Genes

Contigs belonging to ARGs and ERGs were subjected to a taxonomic assignment using Kraken 2 [27] against the Bacterial nr-database [28]. Plots and analyses of taxonomic abundance were made with Pavian-0.3 [29] and MicrobiomeAnalyst [26].

#### 2.3.4. Identification of Mobile Genetic Elements and Their Association with ARGs and ERGs

To determine the ARGs and ERGs presence in mobile genetic elements in the metagenomes, we first determined the presence of plasmids and phages sequences on the metagenomes, and then we evaluated the presence of ARGs and ERGs in these mobile genetic elements. For this, we filtered the contigs to obtain only >1000 pb contigs. Then, Plasflow v1.1 [30] was used for plasmid sequence identification, and DeepVirFinder v1.0 [31] was used for phage sequence detection on the previously described dataset. Afterwards, we identified the ARGs and ERGs in the sequences belonging to mobile genetic elements using the same methodology described in Section 2.3.2. In addition, we performed a taxonomic assignment of the mobile genetic sequences using the same methodology described in Section 2.3.3. Finally, plots and ARGs and ERGs diversity analyses were made using the MicrobiomeAnalyst tool [26].

#### 2.3.5. Co-Occurrence Gene Analysis

Co-occurrence networks were obtained using MetagenoNets [32]. Briefly, NAMAP with Spearman’s rank-correlation algorithm was used to identify positive and negative interactions between ARGs and ERGs. Data were normalized by total sum scaling (TSS), and network correlations with a *p* < 0.05 were considered significant based on bootstrapping of 100 iterations [33]. Finally, Cytoscape v3.9.0 was used for network visualization and FDR corrections [34].

## 3. Results

### 3.1. Sample Collection and Description

Sampling sites in Deception Island are shown in Figure 1. Soils were sampled in a gradient from de Deception hill (1ANT) to the beach zone (5ANT). Sample pH ranged from 6.1 to 7.3, being 1ANT the most acidic sample. Regarding the C and N determinations, all samples showed percentages <1%, except for sample 1ANT, which showed the highest C percentage (3.4%).

### 3.2. Resistance Genes Identification

**Figure 2:**
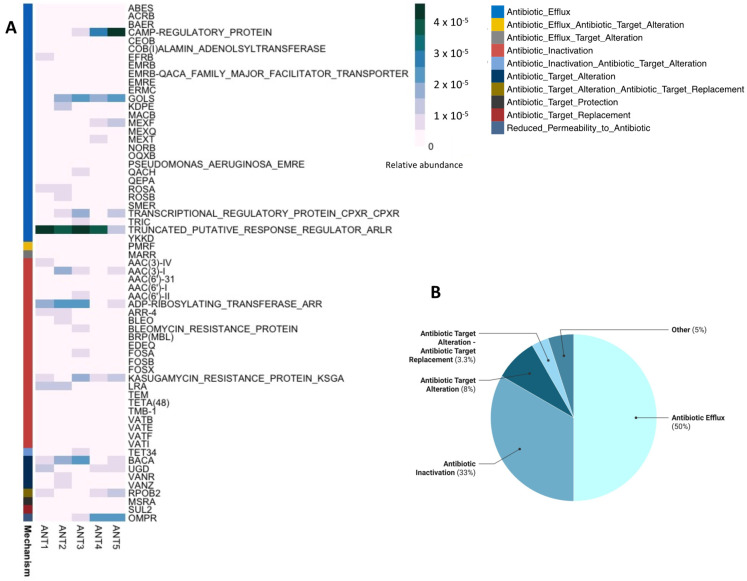
Relative abundance of ARG families in Deception Island (**A**) and their associated resistance mechanisms (**B**). Relative abundance in (**A**) was calculated by dividing the number of ARGs by the total ORFs number per sample. Percentages in (**B**) were calculated as the proportion of each ARGs mechanisms in the total number of detected ARGs.

After quality filtering and trimming steps, 246,138,340 high-quality reads were obtained and assembled, resulting in ~861,000 contigs (>500 pb) per sample. We identified an average of 230,000 open reading frames per metagenome, of which a total of 306 ORFs were classified as ARGs. A total number of 63 ARGs families were identified. The most abundant ARGs were associated with aminoglycosides, beta-lactams, rifamycin, and multidrug resistance genes involving the genes CAMP, GOLS, and ARL (Figure 2A).

The most abundant ARG families among samples were ARLR, GOLS, ARR, and KSGA. Thus, the most abundant resistance mechanisms (Figure 2B and Supplementary Table S1) were Antibiotic Efflux (50%), Antibiotic inactivation (33%), and Antibiotic Target alteration (11.3%).

**Figure 3:**
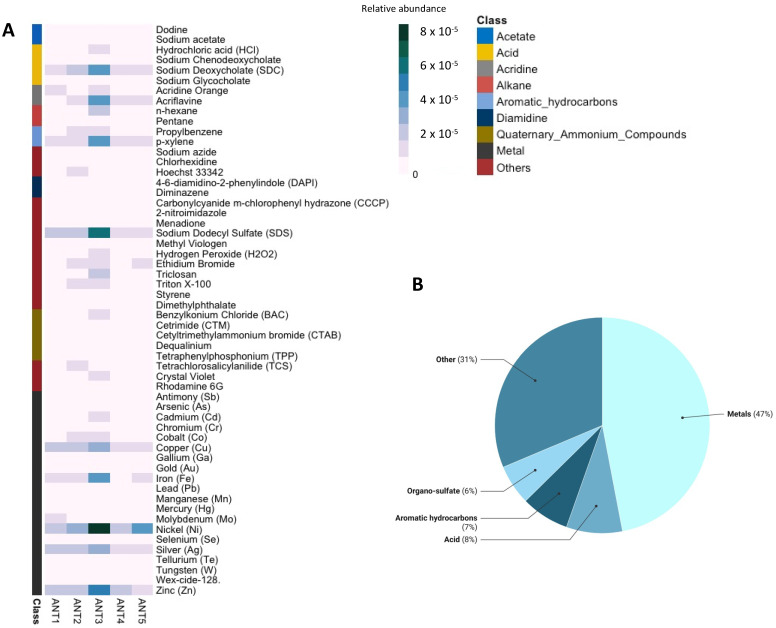
Relative abundance of ERG types in Deception Island (**A**) and their associated resistance class (**B**). Relative abundance in (**A**) was calculated by dividing the number of ERGs by the total ORFs number per sample. Percentages in (**B**) were calculated as the proportion of each ERGs class in the total number of detected ERGs.

On the other hand, we identified 480 ORFs classified as ERGs. A total number of 102 ERGs types associated with metal (47%), acids (8%), and aromatic hydrocarbon (7%) resistance were detected. Specifically, the most abundant were genes associated with Nickel (10%), Cooper (5%), SDC (6%), SDS (5%), and Zinc (6%) resistance (Figure 3A,B).

### 3.3. Taxonomy and Resistance Genes Associations

**Figure 4:**
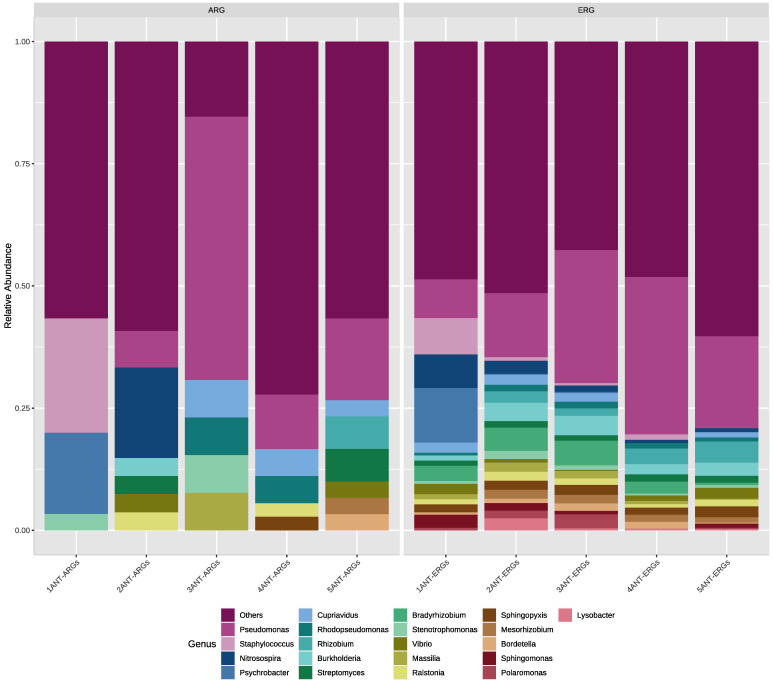
Relative abundance at and genus taxonomic levels for ARGs and ERGs in Deception Island.

The results of ARGs taxonomic annotation (Figure 4) indicated that the most abundant phyla are *Proteobacteria* (75%), *Actinobacteria* (10%), and *Firmicutes* (8%). While the most abundant genera associated with ARGs were *Pseudomonas* (16%), *Staphylococcus* (6%), and *Psycrhobacter* (4%).

Regarding the ERGs, the results indicated that at the phyla level, *Proteobacteria* (84%), *Alphaproteobacteria* (3.9%), and *Actinobacteria* (1.9%) were the most abundant. At the same time, the most abundant genera were *Pseudomonas* (38%), *Burkholderia* (5.5%), and *Bradyrhizobium* (3.7%).

**Figure 5:**
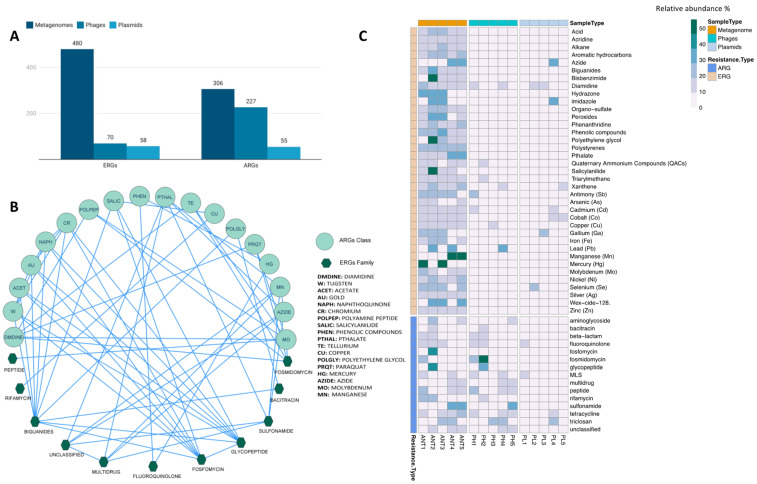
ARGs and ERGs abundance in mobile genetic elements (**A**). ARGs and ERGs positive Co-occurrence network (*p*-value < 0.05) among samples (**B**). Heatmap showing the presence/absence and abundance of ARG and ERG families among the entire metagenome compared to phages and plasmids (**C**). To obtain this presence/absence and abundance heatmap, the abundance of each resistance type was divided by the total sum of each resistance type among metagenome, phages, and plasmids categories.

When analyzing the co-occurrence between ARGs and ERGs in the metagenomes (Figure 5B), it was detected that six classes of ARGs showed a positive co-occurrence (*p* < 0.05) with families of ERGs. These were MLS, Multidrug, Fluoroquinolone, Fosmomycin, peptide, glycopeptides, and unclassified. The results indicate that ERGs of the acid type, peroxides are one of the most important. They may have some co-occurrence relationship with the ARGs of the multidrug and unclassified type, the latter being of great importance since they were the most abundant in the analyzed samples.

On the other hand, molybdenum resistance genes showed co-occurrence with ARGs MLS and peptides. Our results indicate that ERGs associated with Galium and Hydrazona aromatic hydrocarbons showed a direct correlation with ARGs associated with Fluoroquinolones.

### 3.4. Contribution of Mobile Elements to de Dissemination of ARGs and ERGs

Mobile genetic elements were predicted in the metagenomes studied, and results showed a total of 129,530 sequences associated with plasmids and 444,146 with phages. Regarding the resistance genes content (ARGs and ERGs), 113 resistance genes were detected in plasmids, including 58 associated with ERGs and 55 with ARGs (Figure 5A). On the other hand, 297 resistance genes (ARGs and ERGs) were associated with phages, including 70 ERGs and 227 ARGs (Figure 5A). It was determined that ARGs were mainly associated with phages (74%), while ARGs associated with plasmids represent a lower proportion (17.9%) (Figure 5C). Moreover, results indicate that only a small portion of the total number of ERGs is associated with mobile elements since it was found that 14.5% of ERGs were associated with phages and 12% with plasmids (Figure 5C).

On the other hand, only a small portion of ERGs was associated with mobile elements (Figure 5A,B). Thus, it was observed that the most abundant ERGs detected in mobile elements were associated with silver (22%), copper (17%), and zinc (13%). Within the genes associated with phage-type sequences, we find those associated with resistance to azide, imidazole, xanthene, cadmium, cobalt, gallium, and selenium. At the same time, the resistance genes associated with diamidine, quaternary ammonium compounds, antimony, lead (Pb), and cooper were associated with plasmid sequences.

## 4. Discussion

### 4.1. Sample Collection and Description

Studies have reported acidic soils in Deception Island showing even more acidic pH ranges than those detected in our study [14]. Furthermore, high levels of sulfates and oxidizing compounds have been reported in soils, glaciers, and water systems on Deception Island [13].

Regarding the C and N determinations, all samples showed percentages <1%, which could be associated with the geographical origin of Deception Island, which is an active volcano of about 15 km in diameter. In addition, it presents geothermal activity, which generates the existence of hot springs and a large number of fumaroles [14]. On the other hand, Deception Island does not present a great diversity of plants, being the mosses the main representatives. Therefore, there are not many sources of C present in this environment, which may be associated with the low percentages detected in the samples.

### 4.2. ARGs Found in Deception Island

Compared with our results, similar ARGs profiles have been reported in metagenomes from Antarctic soils and extremely cold environments [3,35]. Thus, these resistance gene profiles, belonging to pristine and cold environments, could be considered a good baseline of ARG profiles in low-temperature environments with a low level of anthropogenic impact. Genes associated with rifamycin resistance (ROPB, ARR, and ARR-4) were one of the most abundant in the samples (see Supplementary Table S1). Rifamycin resistance is mainly associated with gram-positive bacteria. However, recent studies have reported that rifamycin resistance is highly prevalent in various bacterial groups [36]. Only one study has reported rifamycin resistance genes in Antarctic ecosystems with low anthropogenic impact [10]. Specifically, these genes were reported in Barrientos Island, Coppermine Peninsula, and Fumarola Bay. On the other hand, the abundance of genes, such as resistance to aminoglycosides, beta-lactams, and multidrug resistance genes, have already been reported in non-intervened environments in Antarctica [3,8,10]. Therefore, the presence of these ARGs in an environment with low anthropogenic intervention, such as Deception Island, could indicate a natural development and fixation of these mechanisms in the bacterial communities of Deception Island.

### 4.3. Resistance Mechanisms in Deception Island

The most abundant resistance mechanisms associated with the ARG profiles (Figure 2B and Supplementary Table S1) were Antibiotic Efflux (50%), Antibiotic inactivation (33%), and Antibiotic Target alteration (11.3%). Antibiotic efflux has been reported as one of the most common resistance mechanisms in Antarctica and in soils from pristine environments in general [3,8,10,37]. In this context, our results indicate that Deception Island’s resistome would also be dominated by these resistance mechanisms.

We found that one of the most abundant ARGs associated with efflux mechanisms was CAMP resistance, which is well known for increasing the virulence of bacterial pathogens and is considered a dangerous ARG in the clinic environment [38]. On the other hand, ArlR and golS, other of the most abundant ARGs in our samples, are known regulators that encode efflux pumps mainly associated with multidrug resistance genes [39,40], which highlight the importance of efflux mechanisms for these bacterial communities. Furthermore, efflux pumps are highly conserved structural components in all members of bacterial species. These proteins are subject to low rates of mutation, which are mainly associated with the specificity of extruded molecules [41]. Therefore, future studies should be focused on these ARGs in order to study the diversity and role of efflux pump families in Deception Island soils.

On the other hand, modification and inactivation mechanisms have been less frequently reported in Antarctic soils. Some modification and inactivation mechanisms have been reported in glacial soils [3,37]. Nevertheless, our results are more similar to those reported by Wei et al., 2015 [9], who described that in Antarctic soil samples, most of the detected ARGs were associated with inactivation mechanisms. In this context, considering that ARR was one of the most abundant inactivation ARGs in our samples, probably one of the most common phenotypic resistance mechanisms in Deception Island soils could be mainly represented by the addition of one or more ADP-ribose moieties to antibiotic molecules [42]. Therefore, attention should be paid to this mechanism concerning the understanding and functionality of antibiotic resistance in Antarctic soils.

Regarding target alteration mechanisms, our results indicate that bacA was one of the most abundant target alteration ARGs. The bacA gene product can bypass the inhibition of the isoprenyl pyrophosphate dephosphorylation caused by bacitracin [43]. It is important to note that this type of resistance is associated with health risks in environmental samples [44]; however, it has been commonly associated with pristine environments [7]. Therefore, its real implications on health risks remain not well studied for this type of environment.

It is important to note that several works have shown that ARGs are the direct drivers of resistance mechanisms in bacterial communities. However, there are situations in which resistance is not driven by a genetic change but is linked to the physiological state of bacteria, nutrients, and environmental stress that microorganisms receive when inhabiting a determined ecosystem [45]. Thus, the presence of ARGs could not determine that these bacterial communities become a risk to environmental health. In this context, the study of the mechanisms of phenotypic resistance is of special relevance in order to understand the role of these ARGs in Deception Island.

### 4.4. ERGs Found in Deception Island

There are few reports in Antarctic metagenomes that describe ERGs associated with arsenic, copper, nickel, chromium, and selenium resistance [10]. However, we have detected very similar ERGs profiles in our samples, and considering the abundance and prevalence, our results suggest that metal resistance genes take special importance for bacterial communities in these soil samples. In this context, studies based on cultivable bacteria showed that the most abundant resistance mechanisms are associated with arsenic, copper, cobalt-zinc-cadmium, and mercury [46].

Our results showed that the abundance and diversity of ERGs were higher than those observed for ARGs. Thus, ERGs likely have adaptative importance for Deception Island bacterial communities, where the presence of metals and oxidative stress is a common feature in these soils [47,48,49]. Finally, the resistance to aromatic hydrocarbons, one of the most abundant ERGs in our samples, draws attention since Deception Island is an environment with low intervention and no record of any contamination event of this type. A recent study reported that ashes from volcanos have high concentrations of aromatic hydrocarbons, which in some cases are high enough to represent a health risk for human health [50]. Considering the fact that Deception Island is an active volcano, it could probably be to find high levels of aromatic hydrocarbons in Deception Island, which could also be one of the environmental factors that propitiate the fixation of these resistance mechanisms in microbial communities. Moreover, the co-occurrence of this type of resistance with an abundance of ARGs associated with efflux pumps has been demonstrated [51], and also studies have reported that aromatic hydrocarbon resistance genes enrich the presence of ARGs in soil samples [51].

### 4.5. Taxonomy and Resistance Genes Associations

Regarding the taxonomy of contigs containing ARGs and ERGs, Van Goethem et al., 2018 [3] reported that the phyla Proteobacteria, Firmicutes, and Actinobacteria were one of the least abundant when analyzing the taxonomy of the detected ARGs. These results differ from the observed in our study in which these phyla could be important contributors to the resistome of Deception Island.

At the genus level, Marcoleta et al., 2022 [10] reported ARGs associated mainly with *Pseudomonas*, *Streptomyces*, *Gemmatimonas*, *Panibacillus*, and *Polaromonas*. In this context, our results indicated that ARGs are mainly associated with the genus *Pseudomonas*, which is consistent with previous reports and highlights the importance of this genus as a reservoir of ARGs. On the other hand, the *Staphylococcus* and *Psychrobacter* genera detected in our study have not been previously reported as relevant genera, in terms of ARGs, in Antarctic metagenomes. However, *Psychrobacter* is a genus known for its resistance to metals and antibiotics [52,53]. On the other hand, *Staphylococcus* is not known as a genus that frequently inhabits this type of environment; however, Vrbovská et al., 2020 [54] reported that animals that inhabit or migrate to Antarctica could be vectors for the dissemination of this bacterial genus. Thus, in future studies, it is important to describe more in-depth the relevance of this genus in Deception Island soils.

On the other hand, regarding ERGs taxonomy, *Pseudomonas* has been described in Antarctic soils as one of the most abundant genera. Moreover, it has shown resistance to environmental stress produced mainly by metals [11,46], which agrees with the results found in our study and highlights the importance of this genus for future studies of environmental resistance in Deception Island.

### 4.6. Co-Occurrence of ARGs and ERGs

When analyzing the co-occurrence, results indicate that ERGs of the acid type and peroxides are one of the most important. They may have some co-occurrence relationship with the ARGs of the multidrug and unclassified type, the latter being of great importance since they were the most abundant in the analyzed samples. Therefore, future studies should give special importance to methodologies for the characterization of these unknown resistance genes, which could give a glimpse into the understanding of the complexity of resistance genes in Deception Island.

On the other hand, molybdenum resistance genes showed co-occurrence with ARGs MLS and peptide. There are several reports where a positive correlation is established between genes for resistance to metals and biocides with ARGs [49,50,51], evidence that has also been recently reported in Antarctic soils by [10]. Our results indicate that ERG genes associated with Galium and Hydrazona aromatic hydrocarbons showed a direct correlation with ARGs associated with Fluoroquinolones. Therefore, considering the characteristics of the soils of Deception Island, where high concentrations of metals [47] and high oxidative stress have been reported, special attention should be paid to how this type of stress may be modulating the presence of ARG genes.

### 4.7. Contribution of Mobile Elements to de Dissemination of ARGs and ERGs

Results associated with mobile elements show the importance of phage-type mobile elements in establishing ARGs in bacterial communities on Deception Island. It is important to note that a large portion of ARGs is associated with beta-lactam, bacitracin, fosmidomycin, multidrug, rifamycin, sulfonamide, triclosan, and unclassified were also associated with mobile phage-like elements, which indicates that this ARGs contained in mobile genetic elements could be eventually disseminated through phages in soils of Deception Island, and therefore future studies should pay special attention to the role of phages in Antarctic soils and their role in the dissemination of resistance genes in the environment.

The importance of phages as ARGs carriers in different environments has been described previously [55,56,57,58,59]. In addition, the importance of phages in the evolution and adaptation of bacterial communities in Antarctica has been described [60,61]. Van Goethem et al., 2018 [3] described that the mobile elements would have a low incidence in the dissemination of ARGs. However, Marcoleta et al., 2022 [10] described the abundance of mobile elements and their direct relationship with ARGs, suggesting that there is a relationship between these mobile elements and the dissemination of that mechanism in the environment. Our results indicate that phages could be one of the main ARGs dissemination mechanisms for the bacterial communities of Deception Island. In addition, this could indicate that the ARGs that are being acquired by the bacterial community are likely modifying bacterial intrinsic functionality. However, the ecological relevance of these ARGs gained through phages needs to be studied in depth. Thus, special emphasis should be placed on the study of mobile elements that may be helping the dissemination of ARGs in the soils of Deception Island.

On the other hand, only a small portion of ERGs was associated with mobile elements. Thus, the low abundance of ERGs in mobile elements may indicate that this type of resistance is part of the intrinsic metabolism of the bacterial communities of Deception Island and that they are essential genes for the establishment of communities in this environment. The fact most ERGs are not associated with mobile genetic elements could indicate that the mechanisms have been fixed through the evolutionary history of these communities, and probably they represent a very specialized metabolic machinery that allows the establishment of these soil bacterial communities. In this context, it is important to note that when resistance is associated with genes on chromosomes, resistant microorganisms will spread more slowly [62].

Moreover, extreme environmental conditions such as biocides, temperature, heavy metals, and other chemical/physical parameters are determinants for the presence and abundance of ARGs and ERGs in bacteria [8]. It is important to note that the ARGs included in phages can be disseminated at a larger temporal and spatial scale than those present in bacterial genomes [63]. Therefore, considering that the ecologic context in Deception Island may be favorable for the dissemination and positive selection of ARGs and ERGs, special attention should be paid to the influence of the environment and phages in the evolution and dissemination of resistance genes in this ecosystem.

## 5. Conclusions

In summary, our results showed the diversity of ARGs and ERGs in Deception Island metagenomes. ARGs of the studied microbial communities are associated with mechanisms such as antibiotic efflux, antibiotic inactivation, and target alteration, which give us a glimpse into the composition of Deception Island soils resistome. On the other hand, resistance to metals, surfactants, and aromatic hydrocarbons could indicate the importance of ERGs for the establishment of microbial communities that inhabit extreme environments such as Antarctica. Furthermore, co-occurrence results may indicate that environmental conditions of this site may have a positive correlation with the prevalence of ARGs in Deception Island soils. Thus, the next studies should give special attention to the relationship between these resistance mechanisms. Results showed the importance of phage-type mobile elements in the establishment of ARGs in bacterial communities on Deception Island. Finally, these results provide valuable information regarding the ARGs and ERGs in Deception Island including the potential contribution of mobile genetic elements to the spread of ARGs and environmental resistance genes in one of the less-studied Antarctic ecosystems to date.

## Figures and Tables

**Table 1:** Coordinates and environmental parameters determination for soil samples.

Sample	Latitude S	Latitude W	pH	%N	%C
1ANT	62°59′0.35″ S	60°32′47.66″ W	6.1	0.48	3.4
2ANT	62°58′57.53″ S	60°33′1.81″ W	6.9	0.1	0.53
3ANT	62°58′57.53″ S	60°33′1.81″ W	7.1	0.77	0.17
4ANT	62°59′15.39″ S	60°33′9.25″ W	7.3	0.13	0.08
5ANT	62°59′10.30″ S	60°33′3.06″ W	6.8	0.01	0.01

## Data Availability

Publicly available datasets were analyzed in this study. This data can be found at https://www.ncbi.nlm.nih.gov (accessed on 8 April 2022) under the accession number provided for each nucleotide sequence.

## Supplementary Materials

The following supporting information can be downloaded at: https://www.mdpi.com/article/10.3390/microorganisms10071432/s1, Table S1: ARGs associated descriptions and mechanisms.
